# Pair-barcode high-throughput sequencing for large-scale multiplexed sample analysis

**DOI:** 10.1186/1471-2164-13-43

**Published:** 2012-01-25

**Authors:** Jing Tu, Qinyu Ge, Shengqin Wang, Lei Wang, Beili Sun, Qi Yang, Yunfei Bai, Zuhong Lu

**Affiliations:** 1 State Key Laboratory of Bioelectronics, Southeast University, Nanjing, 210096, China; 2 Key laboratory of Child Development and Learning Science, Ministry of Education, Southeast University, Nanjing, 210096, China

**Keywords:** barcode, next-generation sequencing, miRNA, breast cancer

## Abstract

**Background:**

The multiplexing becomes the major limitation of the next-generation sequencing (NGS) in application to low complexity samples. Physical space segregation allows limited multiplexing, while the existing barcode approach only permits simultaneously analysis of up to several dozen samples.

**Results:**

Here we introduce pair-barcode sequencing (PBS), an economic and flexible barcoding technique that permits parallel analysis of large-scale multiplexed samples. In two pilot runs using SOLiD sequencer (Applied Biosystems Inc.), 32 independent pair-barcoded miRNA libraries were simultaneously discovered by the combination of 4 unique forward barcodes and 8 unique reverse barcodes. Over 174,000,000 reads were generated and about 64% of them are assigned to both of the barcodes. After mapping all reads to pre-miRNAs in miRBase, different miRNA expression patterns are captured from the two clinical groups. The strong correlation using different barcode pairs and the high consistency of miRNA expression in two independent runs demonstrates that PBS approach is valid.

**Conclusions:**

By employing PBS approach in NGS, large-scale multiplexed pooled samples could be practically analyzed in parallel so that high-throughput sequencing economically meets the requirements of samples which are low sequencing throughput demand.

## Background

Next-generation sequencing (NGS) technologies which are widely employed in life science research, are transforming the biology[1]. In one slide, NGS technologies can generate over one billion DNA sequences now, 1.4 billion by SOLiD 5500 × l (Applied Biosystems Inc.) and 1 billion by Hiseq 2000 (Illumina Inc.). This capacity has increased by dozens of times in the past several years and continuously increases in a rapid speed. The ever increasing reads throughput permits to analyze high complex samples [2,3]. However, for the studies of lower complex samples, such as miRNA discovery, NGS technologies turn to be inefficient and uneconomic. This situation will be aggravated in the future by the ever increasing reads throughput.

In order to relieve the mismatch between the high throughput of NGS technologies and the low throughput requirement of the low complex samples, most NGS technologies provide physically segregated sequencing mediums. But the physical segregation allows accommodation of limited independent samples, and obscures available sequencing space in some NGS technologies[4]. Barcodes, the unique DNA sequence identifiers, historically used in several experimental contexts, are first introduced to the NGS technologies by Meyer et al and Parameswaran et al[4,5] in the pyrosequencing platform. This approach is widely employed to analyze pooled lower complex samples [5-12]. The barcode approach allows the simultaneous analysis of infinite number of samples in theory, but in applications, the researchers analyze at most dozens of samples in parallel[6]. Along with the increasing sample number, the variety of sample-specific primers or adaptors increases at the same speed and this approach becomes less practicable when the sample number becomes large. We have developed a method called pair-barcode sequencing (PBS) for the practical parallel analysis of large-scale multiplexed samples. Although a pair of barcodes was designed to enhance the reliability of experiment[4] or to allow the sequencing starting from both ends of the libraries[10], we have not found any study which assign a pair of barcodes in NGS to improve the practicability and parallelism in analyzing large-scale multiplexed samples.

MicroRNAs (miRNAs) are an evolutionarily conserved class of small, approximately 22-nucleotide (nt) non-coding RNAs that regulate global gene expression patterns[13,14] and play an important role in the development and progression of cancer[15]. Breast cancer, the second-leading cause of cancer related deaths in women, is expected to account for 26% of new cancer diagnoses in 2008[16]. Aberrant expression of miRNAs in human breast cancers were first reported by Iorio et al in 2005[17]. A large number of miRNAs were demonstrated to be associated with breast cancer in the recent years [17-29].

In our method, a pair of molecular barcodes of 6nt in length is designed at the two sides of the target sequences and is introduced to the libraries by adaptor ligation and PCR. We demonstrate the power of PBS by sequencing a pool of 32 human miRNA libraries on the SOLiD V2 platform (Applied Biosystems Inc.). 26 of these libraries were derived from human breast cancer cancerous tissues (BC) and 6 were derived from human breast noncancerous tissues (BO). Four unique forward barcodes (F-barcode) and eight unique reverse barcodes (R-barcode) were used to code the 32 samples. Additionally, we evaluate the correlation of different barcode pairs by performing technical replicates of the same sample with 16 different barcode pairs, and verified the reproducibility of the method by operating two independent sequencing run of the same pool of samples. After decoding and mapping, about 64% of the reads mapped to both of the two barcodes uniquely and 15.3% of decoded reads were identified to non coding RNAs on average. The peak read length of the reads recognized as miRNAs located at 21-22nt, indicating that the mature miRNAs were enriched in the samples and well sequenced. A total of 22 miRNAs were differentially expressed between the BC libraries and BO libraries. To the best of our knowledge, this is the first study to discover miRNAs from large-scale multiplexed samples using a pair of barcodes in NGS.

## Results

### Outline of PBS and design of barcodes

**Figure 1:**
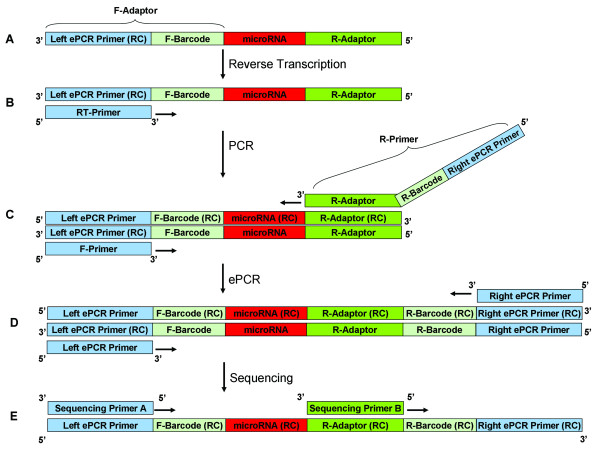
**Outline of PBS**. **A. Ligations**. F-adaptors with F-barcodes at 5' end and R-adaptors are ligated to the miRNAs consequently. **B. Reverse transcription**. Reverse transcription is carried out by RT-primer to form DNA stand. **C. Library amplification**. The libraries were amplified by F-primers and R-primers. The R-barcodes are located in the overhangs at 5' end of R-primers, and the right ePCR primers are devised at the 5' end of R-primers. **D. ePCR**. The ePCR reactions are operated to transfer the libraries to magnetic beads. **E. Sequencing**. The libraries are sequenced by sequencing primer A and sequencing primer B. The sequencing primer A is in charge of sequencing miRNAs and the F-barcodes while the sequencing primer B is responsible for sequencing R-barcodes.

In order to substantially enhance the scope and capacity of multiplexed high-throughput sequencing, a pair of barcodes for 6nt each was designed in two sides of miRNAs. The F-barcodes were devised at the 5' end of the F-adaptors, and were introduced to the libraries by ligating the F-adaptors to miRNAs (Figure 1A). The R-barcodes were designed in the overhangs of the R-primers, and were led to the libraries by PCR reactions (Figure 1C). Each sample was coded by the combination of a unique F-barcode and a unique R-barcode. In order to carry out ePCR reactions, the left and right ePCR primers were designed in the pair-barcoded library. The left ePCR primers (reverse complementary sequence) were located at the 3' end of F-adaptors and the right ePCR primers were designed in the overhangs of the R-primers, at the 5' end the R-barcodes. Two sequencing primers, sequencing primer A and sequencing primer B, were used to execute the sequencing reactions. The sequencing primer A is in charge of sequencing the F-barcodes and miRNAs while the sequencing primer B is responsible for sequencing R-barcodes. The sequence of each reads was obtained after sequencing runs and is made up of 3 parts, the F-barcode, the R-barcode and the target sequence. By decoding the F-barcodes and R-barcodes consequently, the target sequences were ascribed to the different samples.

As one mismatch is tolerated in barcode mapping, it is impossible to get uniquely mapped reads if all permutations of 6 nucleotides are allowed to be barcodes. In order to get uniquely mapped reads, each barcode should be different with each other in at least three positions. Moreover, because the mapping of SOLiD reads is operated in the color space, the barcodes are designed to be different with each other in at least two positions in the color space, not in the nucleotide space.

### Pair-barcode decoding

After the sequencing reactions, 89175789 barcoded sequences were obtained in the sequencing run I, while 85344522 barcoded sequences were obtained in the sequencing run II. 71.3% and 70.4% of the obtained barcoded sequences were mapped to the 4 F-barcodes used in the libraries generation, respectively. And 89.7% and 91.4% of the F-barcode mappable sequences were mapped to the 8 R-barcodes introduced in the PCR reactions, separately. In total, 64.0% and 64.4% of the obtained sequences were mapped to a used F-barcodes and a used R-barcodes at the same time. The reads mapped to each combination of F-barcodes and R-barcodes are counted and exhibited in Table 1.

**Table 1:** Decoded read counts and raw miRNA expression values

Barcode pair/dataset	Pilot run I	Pilot run II
A1	1350957	1248344
A2	839729	812507
A3	654378	632717
A4	1558780	1516755
A5	1106683	1059838
A6	1083017	1086001
A7	2413058	2464352
A8	3449159	3397171
B1	743743	679503
B2	236758	225889
B3	172964	163368
B4	236646	231825
B5	585148	555769
B6	1315460	1276797
B7	710279	711801
B8	1633113	1565130
C1	12646912	11782099
C2	2708545	2635196
C3	1623237	1556327
C4	1537402	1521985
C5	928983	879860
C6	721715	742206
C7	2326266	2372511
C8	2721671	2651552
D1	1536539	1381501
D2	432199	403448
D3	2670887	2529404
D4	2770368	2662417
D5	482720	453340
D6	1750057	1707542
D7	1193768	1201293
D8	2552421	2464702

### Mapping results

Composition of each library was determined by filtering all decoded reads to human genome. MiRNAs were identified by mapping reads to pre-miRNAs in miRBase and quantified by mapping reads to mature miRNA in miRBase. About 46% of the decoded sequences were mappable using SOLiD system small RNA analysis pipeline tool (RNA2MAP, version 0.5.0) (Additional file 1). Unmappable sequences were neither other non-coding human RNAs, nor fragment of human genome, as they passed the filtering of other non-coding human RNAs and did not map to the human genome.

**Figure 2:**
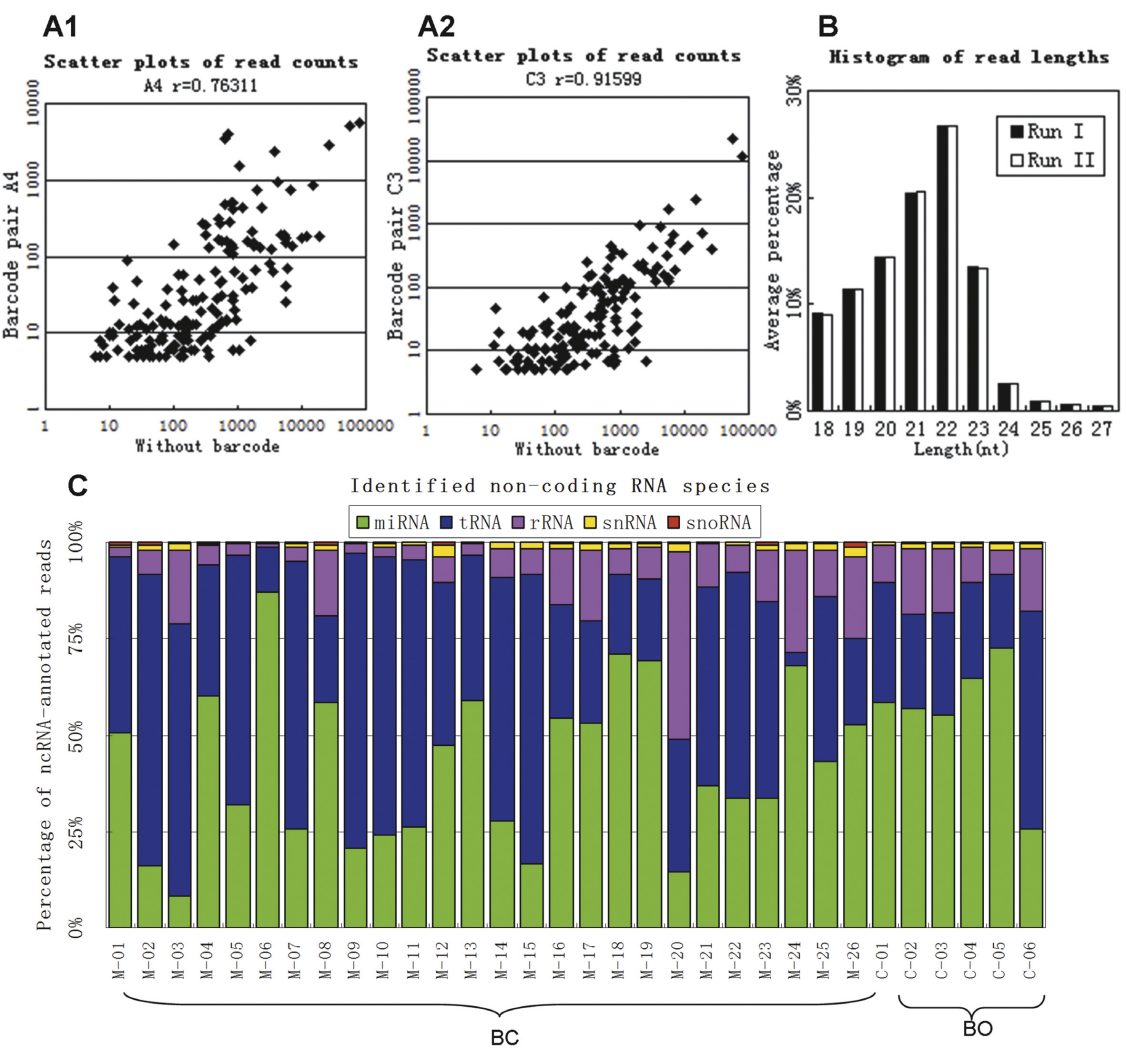
**NGS of the small RNA transcriptome**. **A1**. Scatter plots of raw miRNA expression values of the same sample with the worst correlation. The x-axis was determined without barcode and the y-axis determined with barcode pair A4. **A2**. Scatter plots of raw miRNA expression values of the same sample with the best correlation. The x-axis was determined without barcode and the y-axis determined with barcode pair C3. Raw expression values of miRNAs determined with 16 kinds of barcode pairs were compared with raw expression values of miRNAs without barcode one by one, and the scatter plots are exhibited in Additional file 4. **B**. Read length distribution (nt) of known miRNAs by removing adaptors and barcodes. The *y*-axis depicts the percentage of read lengths relative to the total number of reads in all datasets. Read length distributions of each patient are shown in Additional file 2. **C**. Distribution of non-coding RNA species in the all 32 samples.

After adaptor and barcode sequence removal, read length distribution of the reads mapping to the miRBase revealed a peak at 21-22nt (Figure 2B, Additional file 2), indicating that mature miRNAs were enriched in the sequenced samples. In order to evaluate the consistency of the miRNA expression values of the two independent runs, the raw expression values of miRNA were compared in on logarithmic coordinate. Scatter plots of each sample revealed a well reproduction quality in miRNA expression, especially in the miRNAs whose expression values (read counts) over 5 (Additional file 3). The expression values were calculated by counting the reads which mapped to the mature miRNAs in miRBase. On average, about 15.3% (range 6.1%-38.6%) of all decoded reads were identified as non-coding RNA. MiRNA was the major constituent among the non-coding RNA, ranged from 10% to 85%, and tRNA was the other major non-coding RNA species (Figure 2C, Additional file 1). The proportion of decoded reads mapped to other non-coding RNA, miRNA, and genome was different across the datasets (Additional file 1). No significant variation of miRNA proportion between breast cancerous tissue datasets and breast noncancerous tissue datasets was detected.

### Correlation of miRNA read counts without barcode to miRNA read counts with different barcode pairs

To validate sequencing data with pair-barcode sequencing approach, we examined the correlation of raw read counts between dataset without barcode and datasets with 16 different barcode pairs. The miRNAs whose raw read counts were > 5 in both datasets for comparison were selected to calculate the Pearson's correlation coefficient. Expression of most miRNAs was highly correlated between the technical replicates. Comparing with the results without barcode, the Pearson's correlation coefficients ranged from 0.76 to 0.92 for each barcode pair (Figure 2A, Additional file 4). We conclude that raw read counts obtained from pair-barcode sequencing are valid and strong correlation to results obtained without barcode.

### High expressed miRNAs

We first analyzed the high expressed miRNAs both in BC datasets and BO datasets. The miRNAs whose normalized expression value over 100 in at least 20 datasets of the all 25 datasets (19 BC datasets and 6 BO datasets) were shown in Additional file 5 Table S5 (Additional file 6 Additional file 5). These criteria were met by 85 miRNAs, including 5 miRNA-3p sequences and 6 miRNA-5p sequences. But no miRNA* met these criteria. The 10 most abundantly expressed miRNAs in both kinds of datasets are exhibited in Table 2. The average of BC was divided by the average of BO and the quotient was taken the logarithm to calculate the ratio, based on 2. Three of these 10 miRNAs, miR-21, let-7b, and miR-19b, expressed differently between BC datasets and BO datasets (ratio<-1 or ratio > 1).

**Table 2:** Highly expressed miRNAs in all datasets.

	Average read counts		Ratio
	BC	BO	
miR-23a	37,714	39,969	-0.08
miR-21	20,007	6,091	1.72
miR-19b	16,705	7,368	1.18
let-7b	7,871	33,016	-2.07
miR-27a	12,514	16,534	-0.40
miR-1308	11,871	12,091	-0.03
miR-181a	10,825	8,456	0.36
miR-29a	10,558	9,074	0.22
miR-205	10,346	7,839	0.40
miR-1975	7,876	6,044	0.38

The average of BC was divided by the average of BO and the quotient was taken the logarithm to calculate the ratio, based on 2.

### Analysis of known breast cancer related miRNAs

**Figure 3:**
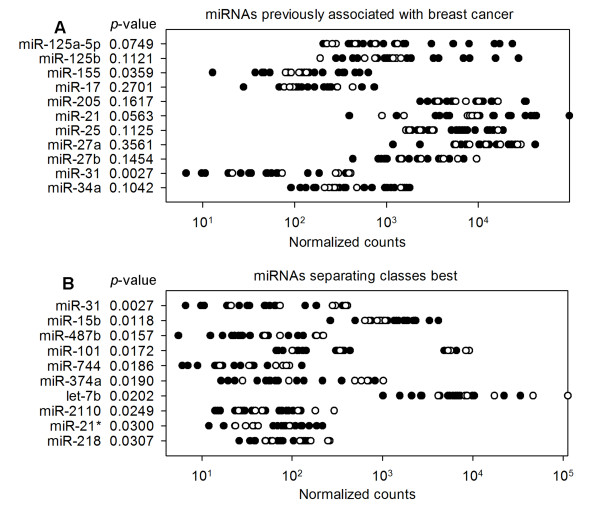
**Differential miRNA expression between BC and BO**. The expression value zero was set as one to be visible on logarithmic coordinates. The normalized expression values were used to evaluate the difference between two kinds of datasets. Black spots refer to breast cancer cancerous tissue datasets and black circles depict breast noncancerous tissue datasets. **A**. Expression data for known breast cancer associated miRNAs. **B**. Expression data for the 10 best class-separating miRNAs. Rows are sorted according to raw *p*-values. These data including a previously breast cancer associate miRNA (shown in Figure 3A) as well as newly identified differentially expressed miRNAs.

We also analyzed miRNAs previously reported to be differentially expressed in BC, including oncogenic miRNAs, miR-155[17], miR-21[18,19], miR-25[30], and miR-27a[24], and tumor suppressor miRNAs, miR-125a/b[17], miR-17[26], miR-205[25], miR-27b[24], miR-31[27], and miR-34a[28,29]. The expression values of these miRNAs between BC datasets and BO datasets were compared in logarithmic coordinates (Figure 3A). NGS using pair-barcode revealed that the oncogenic miRNA of miR-155 which were previously shown to be upregulated in tumors [17-19], were also upregulated in BC datasets comparing to the BO datasets. The tumor suppressor miRNA of miR-31 were downregulated in BC datasets comparing to the BO datasets [27]. The other breast cancer associated miRNAs did not exhibit significant difference between BC datasets and BO datasets in our study.

### MiRNAs differentially expressed between two clinical groups

We aimed to identify other miRNAs differentially expressed in BC datasets versus BO datasets using pair-barcode NGS. Only mature miRNAs represented by more than 5 raw counts in at least 20 datasets of all 25 datasets were considered. 200 miRNAs met these criteria, including 18 star miRNA, 20 miRNA-3p and 20 miRNA-5p sequences. A total of 22 miRNAs were differentially expressed based on a *t*-test (Additional file 7), including 1 miRNA*, 3 miRNA-3p and 1 miRNA-5p sequences. Figure 2B exhibits the top 10 differentially expressed miRNAs, including miRNAs previously associated with BC, miR-31.

## Discussion

PBS allows for sequencing large-scale multiplexed samples using SOLiD system and any other NGS platforms. Comparing to the physical segregation of the sequencing plate, PBS permits to parallel sequencing much larger amount of samples. In some NGS platforms, physical segregation lessens the available throughput by obscuring available sequencing space. The lessening of throughput is also found in PBS approach because the barcodes shortens the available read length. But for some studies, such as miRNA expression studies, the read count is more significant than the read length when the read length scarified the requirements of these studies. The read length after removing 6nt F-barcodes is 29nt in our study, which is longer than most mature miRNAs. Furthermore, the available read length of the latest NGS platforms are longer than the SOLiD V2 system, 75nt of SOLiD 5500 × l and 100nt of Hiseq 2000. PBS is more economic and efficient than the physical segregation in these applications. In the existing single barcode method which allows parallel analysis infinite samples in theory like PBS, the number of tagged oligos required is equal to the number of the samples pooled together. If the number of pooled samples is large, the same number of tagged oligos is required and the single barcode method lost its practicability. PBS approach is considered to be an ideal method to overcome this problem. The latest sequencers generate up to 1 billion reads, which is adequate to parallel analysis up to 100 miRNA samples. To parallel analyze 100 miRNA samples, single barcode method requires 100 barcoded oligos (Table 3). By combining with physical segregation, at least 13 barcoded oligos are remaining required. However, totally 20 barcoded oligos are necessary by PBS without the help of physical segregation. The throughput of NGS has increased for dozens of times in the past several years and continuously increases in a rapid speed. The superior of PBS will be more significant when the throughput becomes larger. For example, if the researchers want to parallel analyze 10,000 samples in the future, 10,000 kinds of sample-specific primers or adaptors are needed by the existing single barcode method. Combining with physical segregation, 1250 kinds of barcoded oligos are still indispensible. However, only 100 varieties F-adaptors and 100 sample-specific R-primers are required using the PBS approach (Table 3). Moreover, F-adaptors and R-primers are employed in two different steps of library construction, reducing the probability of potential mistakes made within the library construction. Comparing to the existing single barcode method, PBS approach requires significantly less kinds of tagged oligos, about square root of the single barcode method. Therefore, the studies become uncomplicated and efficient.

**Table 3:** Comparison of the current methods versus PBS.

Samples number	PSS ^1^	SBS ^2^	Combination of PSS ^1 ^and SBS ^2^	PBS
10	Allowing up to 8 samples	10 barcoded oligos	4 segments with 3 barcoded oligos	7 barcoded oligos (4 F-barcodes and 3 R-barcodes)
100	N/A	100 barcoded oligos	8 segments with 13 barcoded oligos	20 barcoded oligos (10 F-barcodes and 10 R-barcodes)
1000	N/A	1000 barcoded oligos	8 segments with 125 barcoded oligos	67 barcoded oligos (34 F-barcodes and 33 R-barcodes)
10000	N/A	10000 barcoded oligos	8 segments with 1250 barcoded oligos	200 barcoded oligos (100 F-barcodes and 100 R-barcodes)

^1 ^PSS, Physical Space segregation

^2 ^SBS, Single barcode sequencing

About 71% of the reads mapped to the sequence of the 4 F-barcodes and about 90% of the F-barcode decoded reads mapped to the sequence of the 8 R-barcodes. In each of the two decoding steps, 20% of the reads are off-target on average (Figure 2C). The ratios are logical comparing to other SOLiD works [14]. The vast majority of these off-target reads did not mapped to both of the two barcodes, indicating that they were not caused by using a pair of barcodes instead of a single barcode. The counts of reads mapped to the barcode pairs are tens of times difference between each other. The existing DNA spectrophotometers, such as Nanodrop ND-1000 are not fit for determining samples whose concentration smaller than 2 ng/μl directly. The final concentration of pooled libraries used for ePCR is 50 pg/μl. The libraries were determined at high concentration and were diluted to 50 pg/μl. The dilution may be one of the reasons of read counts difference cross datasets. Although the read counts difference induced the difference in miRNA expression, this difference can be resolved by read counts normalization.

The percentage of mappable reads using SOLiD system small RNA analysis pipeline tool (RNA2MAP, version 0.5.0) is at the same level of other work [14]. The read length distribution after mapping to the miRBase is identical with the other studies [14,31], indicating that mature miRNAs were enriched in the sequenced samples and were well sequenced by the PBS. Hundreds of miRNAs discovered in the pooled samples is another support. We found an average correlation of r = 0.999 between miRNA expression counts in replicate experiments, indicating the sequencing assay is highly reproducible.

Quantile-quantile normalization was used to normalize raw read counts to remove potential bias in miRNA expression across the datasets. The quantile-quantile normalization has been used to normalize the raw read counts of NGS and shown to be superior to scaling to a given constant [14]. We first analyze the high expressed miRNAs (Table 2). Three of these 10 miRNAs expressed differently between BC datasets and BO datasets (ratio<-1 or ratio > 1). miR-21, one of the two upregulated miRNAs, is a well known oncogene that expected to be abundant in breast tumor [18,19]. miR-19b, the other upregulated miRNA, is reported to be high expressed in invasive breast cell lines versus less invasive breast cell lines[23]. The downregulated miRNA, let-7b, is a member of let-7 family which considered to be tumor suppressor miRNAs [20].

We then analyzed miRNAs that previously associate to breast cancer. MiR-155, which was discovered to be upregulated in breast cancer [17-19], is also significant upregulation in BC datasets, suggesting that it may play as an oncogene in breast cancer. MiR-31, which is expressed in normal breast cells and was recently shown to prevent metastasis at multiple steps by inhibiting the expression of prometastatic genes [27], was observed to be downregulated in BC datasets. No significant difference between BC and BO datasets was observed in the other 9 breast cancer associate miRNAs.

We also analyzed other miRNAs differentially expressed between BC and BO datasets in this work. Of all the 200 miRNAs considered, 22 miRNAs were differentially expressed according to the *p*-values. We compared the expression patterns of the 10 differentially expressed miRNAs with published results (Figure 3B). MiR-31, downregulated in BC datasets, was reported to be a suppressor of breast cancer. MiR-21* was upregulated in BC datasets and the non star form of miR-21* was recognized as a breast cancer oncogenic miRNAs [18,19]. As a member of let-7 family, a well known breast cancer suppressor, let-7b was observed to be downregulated in BC datasets in our study. Several miRNAs among the 10 most differentially expressed miRNAs were associated other malignancies in published studies, including miR-15b[32,33], miR-101[34], miR-374a[35], and miR-218[36]. The expression patterns of these 4 miRNAs in our study are all in accord with the work of profession. This accordance strengthens that these 4 miRNAs are breast cancer concerning miRNAs. We here report that miR-15b, miR-101, miR-374a and miR-218 are breast cancer associated miRNAs by the sequencing results of our PBS approach. For the rest 3 miRNAs, miR-487b, miR-744 and miR-2110, no paper was found to report any relationship with malignancies. We notice that all these 3 miRNAs have low read counts in the two divergent clinical groups, indicating low expression values in tissues. It may be the reason why the different expression patterns of these miRNAs were not reported in previous works.

## Conclusions

In summary, we have developed an ideal system that allows for high-throughput sequencing of large-scale multiplexed samples using SOLiD system. Substantially, comparing to the existing barcode sequencing, the supposed technique improves the practicability when the amount of samples is large. The application demonstrates that PBS is a valid tool to discover miRNAs in large-scale multiplexed samples. PBS approach can be readily adapted to any existing NGS platform and will be more widely applied along with the increasing of NGS throughput in the next several years.

## Methods

### Sample preparation and RNA isolation

All participants provided written informed consent, and the study received ethical approval from the ethics committee of the State Key Laboratory of Bioelectronics. 32 samples were derived from 26 human breast cancer cancerous tissues (M1-M26) and 6 human breast noncancerous tissues (C1-C6) and kept in TRIzol (Qiagen Inc., Shanghai, China) before RNA isolation. MiRNAs were extracted from each sample using mirVana miRNA isolation kit (Ambion Inc., Austin, TX, USA). MiRNA quantity and quality were checked by spectrophotometer, Nanodrop ND-1000 (Thermo Fisher Scientific Inc., Waltham, MA, USA). The clinical data of patients are shown in Additional file 8.

### Small RNA library generation and sequencing

F-adaptors and R-adaptors were ligated to miRNA samples consequently with a size-selection by polyacrylamide gel electrophoresis to purify the library after each ligation. Each F-adaptor contains a unique F-barcode for 6-nt in length. The RT-primers were added to carry out reverse transcription. The libraries were then amplified by PCR reactions, in which the R-barcodes for 6-nt in length were introduced by the R-primers. One more size-selection by polyacrylamide gel electrophoresis was done after the PCR reactions. The 32 samples were mixed in a pool at the same concentration to operate emulsion PCR (ePCR). Template bead preparation, ePCR and deposition were performed according to the standard protocol, and slides were analyzed on a SOLiD system V2 (Applied Biosystems Inc., Foster City, CA, USA) according to the multiplexing protocol. The sequences of oligos used in this paper are shown in Additional file 9. Sample M-14 was selected to perform technical replicates with different barcodes. This sample was sequenced directly without barcode, and with 16 barcode pairs.

### Decoding, mapping and data analysis

SOLiD reads were decoded by the F-barcodes and R-barcodes consequently allowing one mismatch in each 6nt coding region. The reads which match an F-barcode and an R-barcode uniquely and simultaneously were used for mapping (Additional file 9). Mapping of SOLiD reads was performed using SOLiD system small RNA analysis pipeline tool (RNA2MAP, version 0.5.0). After filtering the other human non-coding RNAs, raw expression values (read counts) were obtained by summing the number of reads that mapped uniquely to the reference database, miRBase release 14.0 and Human Genome RefSeq Hg19. We allowed two mismatches for the first 18nt and three mismatches for the remaining 11nt of each decoded reads (Additional file 6).

### Normalization of read counts

In order to quantify and compare miRNA expression across datasets, raw read counts were normalized using quantile-quantile normalization to remove a potential bias. Dataset M1 was chosen as a reference, and the scaling factors were obtained by computing the median of differences of corresponding quantile values of a dataset and the reference dataset. The distribution of absolute count values > 5 both in the dataset and the reference dataset were compared in logarithmic space. All datasets were normalized by linear transformations using the scaling factors (Additional file 6) [14].

## Abbreviations

BC: breast cancer cancerous tissues; BO: breast noncancerous tissues; F-adaptor: forward adaptor; F-barcode: forward barcode; MiRNA: microRNA; NGS: next generation sequencing; nt: nucleotide; PBS: pair barcode sequencing; ePCR: emulsion PCR; PSS: Physical Space segregation; R-adaptor: reverse adaptor; R-barcode: reverse barcode; SBS: Single barcode sequencing; TNList: Tsinghua National Laboratory for Information Science and Technology.

## Authors' contributions

JT conceived of the study, participated in its design, coordination and data analysis and drafted the manuscript. QG conceived of the study, participated in its design, coordination and drafted the manuscript. SW and LW performed the data analysis. BS participated in the design and participated in data analysis. QY participated in the sequencing experiment design. YB conceived of the study, participated in its design. ZL conceived of the study participated in its design and coordination, and helped to draft the manuscript. All authors read and approved the final manuscript.

## Supplementary Material

Additional file 1 **Statistics after mapping**. Statistics after mapping decoded NGS reads to the other non coding RNAs, Human Genome (RefSeq Hg19) and miRBase (Release 14.0). Click here for file

Additional file 2 **MiRNA read length distribution**. Exhibit the miRNA read length distribution of each patient. Click here for file

Additional file 3 **MiRNA read counts of two independent runs**. Scatter plots of miRNA expressions of all 32 barcode pairs of two independent runs. Click here for file

Additional file 4 **MiRNA read counts of different barcode pairs**. Scatter plots of miRNA read counts of the same sample without barcode to with different barcode pairs. Click here for file

Additional file 5 **Highly expressed miRNAs**. Highly expressed miRNAs in all 25 datasets. Click here for file

Additional file 6 **Mapping and normalization**. The detailed progression of mapping and normalization. Click here for file

Additional file 7 **Different expressed miRNAs**. The 22 miRNAs which were differentially expressed between breast malignant and breast carcinoid based on a *t*-test. Click here for file

Additional file 8 **Clinical data**. Clinical data of the patients included in this study. Click here for file

Additional file 9 **Encoding and decoding**. Details of the encoding and decoding progress. Click here for file
