# Supplementary material for: Pair-barcode high-throughput sequencing for large-scale multiplexed sample analysis
Source: BMC Genomics. 2012 Jan 25;13:43. doi: 10.1186/1471-2164-13-43 (PMC3284879; doi:10.1186/1471-2164-13-43)
Supplement: Additional file 1 — Statistics after mapping. Statistics after mapping decoded NGS reads to the other non coding RNAs, Human Genome (RefSeq Hg19) and miRBase (Release 14.0). [file 1471-2164-13-43-S1.PDF]

Additional file 1, Statistics after mapping.

Statistics after mapping decoded NGS reads to the other noncoding RNAs, Human Genome (RefSeq Hg19) and miRBase (Release 14.0). We allowed two mismatches for the first 18nt and three mismatches for the remaining 11nt of each decoded reads. Rows represent datasets A1-D8. Row "Average" shows the unweighted average statistics. Column "Sequencing Run I" and "Sequencing Run II" represent the statistics obtained the two pilot runs separately. Column "filter" shows the percentage of reads mappable to the non-coding RNA library (not including miRNA). Column "miRNA" shows the percentage of reads mappable to the miRBase. Column "genome" shows the percentage of reads mappable to the human genome (excluding to the reads mappable to the non-coding RNAs). The final column "nomap" lists the percentage of reads that could not be mapped.

	Sequencing Run I				Sequencing Run II			
Dataset	filter	miRNA	genome	nomap	filter	miRNA	genome	nomap
A1	12.6%	12.8%	31.6%	42.9%	11.9%	12.5%	31.8%	43.8%
A2	19.4%	3.7%	33.9%	43.0%	17.9%	3.5%	33.4%	45.1%
A3	19.1%	1.8%	33.0%	46.2%	18.1%	1.6%	33.1%	47.2%
A4	4.2%	6.1%	13.0%	76.7%	3.8%	6.0%	13.5%	76.6%
A5	23.4%	10.6%	21.4%	44.5%	21.8%	10.4%	22.3%	45.4%
A6	3.1%	20.1%	29.9%	46.9%	2.7%	19.0%	30.2%	48.2%
A7	28.8%	9.8%	21.9%	39.5%	27.1%	9.5%	22.3%	41.2%
A8	2.7%	3.7%	25.7%	68.0%	2.5%	3.6%	25.8%	68.1%
B1	5.3%	1.4%	34.0%	59.3%	5.1%	1.3%	33.7%	59.9%
B2	10.1%	3.1%	36.9%	49.9%	9.1%	3.0%	36.1%	51.8%
B3	9.6%	3.4%	35.9%	51.1%	9.4%	3.3%	36.0%	51.3%
B4	4.4%	3.9%	34.3%	57.4%	3.9%	3.5%	33.2%	59.3%
B5	6.4%	9.0%	33.0%	51.6%	5.7%	8.5%	32.4%	53.3%
B6	12.9%	4.9%	34.8%	47.4%	12.0%	4.7%	34.1%	49.1%
B7	12.1%	2.4%	41.1%	44.5%	11.0%	2.3%	40.3%	46.5%
B8	1.7%	2.2%	24.6%	71.5%	1.5%	2.2%	23.4%	72.9%
C1	6.0%	7.7%	34.5%	51.7%	5.8%	7.7%	34.0%	52.5%
C2	6.6%	7.8%	36.0%	49.7%	6.3%	7.6%	35.4%	50.7%
C3	7.1%	8.6%	36.3%	48.1%	6.9%	8.6%	36.0%	48.6%
C4	5.8%	6.4%	35.3%	52.4%	5.3%	6.1%	34.6%	54.0%
C5	4.1%	9.8%	35.6%	50.6%	3.8%	9.5%	34.7%	52.0%
C6	3.8%	6.7%	35.0%	54.5%	3.3%	6.3%	34.3%	56.1%
C7	5.1%	13.3%	35.8%	45.7%	4.7%	12.9%	35.1%	47.2%
C8	4.5%	10.1%	33.5%	51.8%	4.3%	10.0%	32.9%	52.8%
D1	9.7%	3.3%	26.0%	61.0%	9.6%	3.4%	26.6%	60.5%
D2	9.8%	1.7%	33.1%	55.4%	9.0%	1.5%	33.2%	56.4%
D3	8.8%	5.1%	15.8%	70.3%	8.8%	5.1%	15.7%	70.4%
D4	10.1%	5.0%	15.9%	69.0%	9.6%	4.9%	16.2%	69.3%
D5	12.5%	6.2%	31.2%	50.1%	11.8%	6.1%	31.1%	50.9%

D6	2.5%	5.2%	41.6%	50.6%	2.3%	4.9%	40.6%	52.2%
D7	7.3%	5.5%	22.5%	64.7%	6.9%	5.3%	22.4%	65.3%
D8	5.5%	5.9%	36.8%	51.8%	5.1%	5.8%	36.4%	52.7%
Average	8.9%	6.5%	30.9%	53.7%	8.3%	6.3%	30.6%	54.7%
