# Supplementary material for: Pair-barcode high-throughput sequencing for large-scale multiplexed sample analysis
Source: BMC Genomics. 2012 Jan 25;13:43. doi: 10.1186/1471-2164-13-43 (PMC3284879; doi:10.1186/1471-2164-13-43)
Supplement: Additional file 2 — MiRNA read length distribution. Exhibit the miRNA read length distribution of each patient. [file 1471-2164-13-43-S2.PDF]

Additional file 2, MiRNA read length distribution.

MiRNA read length distribution of each patient. The top row identifies the dataset. The leftmost column identifies the sequencing run, and the next column identifies the read length. Numbers in the table specify the number of decoded reads of each length in each dataset after mapping to the miRBase and adapter removal.

	Length	A1	A2	A3	A4	A5	A6	A7	A8
	18	22020	1717	1543	2036	2628	53654	6009	47811
	19	20163	2066	1519	3226	4509	43779	10591	34906
	20	42989	5564	2321	9221	6867	47923	16943	25118
	21	43321	10849	2762	25117	18585	36932	46102	22225
Sequencing	22	34309	6036	2852	36308	54599	26546	105843	16689
Run I	23	15501	3639	1696	16507	25299	13333	43566	9022
	24	3174	946	413	3469	6991	2455	9636	1991
	25	1138	899	352	1012	1723	1084	2918	881
	26	777	1110	408	802	572	281	1641	673
	27	292	861	277	352	676	131	904	1269
	18	19988	1569	1350	1982	2348	50615	5759	45745
	19	18213	1919	1396	3114	4325	41487	10566	33393
	20	38452	4886	2073	8993	6336	45716	17112	24109
	21	40074	10303	2422	24338	17288	35138	45718	21381
Sequencing	22	30362	5357	2513	34522	50950	25039	105026	16511
	23	14422	3323	1545	15925	24188	12497	43826	8749
Run II	24	3011	834	382	3294	6661	2365	9319	1990
	25	1067	790	291	949	1494	921	2627	860
	26	610	973	370	646	476	258	1342	623
	27	302	773	205	286	610	96	787	1164

	Length	B1	B2	B3	B4	B5	B6	B7	B8
	18	1579	701	615	2607	6734	11321	1039	4630
	19	1900	927	696	1583	9196	13275	1354	5490
	20	1997	1592	760	1658	10551	12573	1993	6800
	21	1458	1103	788	1234	10496	12482	2284	6402
Sequencing	22	2147	1565	1407	1178	13822	10778	4564	8787
Run I	23	1762	1562	1508	1092	2599	7591	4676	5451
	24	222	224	149	221	638	1663	702	534
	25	200	135	127	157	457	1171	1012	208
	26	200	139	161	100	305	839	1183	203
	27	160	75	42	47	286	1307	300	157
	18	1459	623	608	2291	5985	10156	958	4433
Sequencing	19	1760	904	619	1426	8544	12878	1301	5337
	20	1712	1495	780	1579	9590	11946	1990	6351
Run II	21	1273	1099	727	1103	9382	11773	2239	6207

	22	1839	1486	1375	1063	12543	10149	4551	8360
	23	1506	1207	1344	892	2249	6816	4215	5125
	24	166	229	120	196	564	1554	630	506
	25	154	128	92	140	411	1203	876	187
	26	177	147	149	65	263	817	1058	169
	27	126	76	34	38	205	1211	241	120

	Length	C1	C2	C3	C4	C5	C6	C7	C8
	18	98867	23814	14354	9662	8198	5138	18903	21351
	19	137266	34719	19409	15349	12252	6905	32041	34429
	20	158553	35801	21912	16527	14235	8084	42660	43888
	21	200819	38231	29045	20115	19578	11562	79007	63615
Sequencing	22	254385	52547	36037	24848	23185	12461	94498	71229
Run I	23	138922	26794	19245	13243	11725	4992	39175	35731
	24	13902	2997	2081	1493	2336	779	7413	6879
	25	6132	1177	800	758	1185	239	2218	2793
	26	3857	959	591	510	547	181	1631	1116
	27	4042	724	397	533	676	149	1339	1999
	18	89671	22238	13570	8997	7661	4867	18325	20561
	19	127984	33306	18566	14780	11454	6884	31641	32844
	20	147668	33647	20783	15513	13138	7830	41891	42401
	21	188120	36628	27994	19001	17788	11296	78956	61756
Sequencing	22	237432	50146	34897	23533	21205	11995	92899	68563
	23	129133	24980	18553	12371	10721	4836	38586	34505
Run II	24	13315	2759	2024	1418	2268	743	7446	6633
	25	5456	1132	701	648	1080	232	2056	2619
	26	3557	862	508	467	523	141	1428	1096
	27	3567	657	347	410	582	115	1243	1821

	Length	D1	D2	D3	D4	D5	D6	D7	D8
	18	1433	632	3367	2721	895	5033	1903	6036
	19	2385	827	4498	4457	1424	7265	3079	9700
	20	4744	1013	11869	11299	3249	17793	8600	17483
	21	10161	1712	47432	25038	6138	25774	16040	29766
Sequencing	22	17796	2069	48678	48561	10982	34339	22896	51363
Run I	23	11499	1164	19499	37657	6259	7638	11684	30153
	24	3570	749	4126	9808	1931	1309	3305	11576
	25	1413	312	665	2632	535	360	1259	4225
	26	539	184	317	861	270	397	793	3386
	27	475	84	249	905	100	141	237	634
	18	1270	515	2979	2439	864	4300	1869	5615
	19	2142	752	4245	4053	1422	6688	3066	9075
Sequencing									

Run II	20	4313	828	11455	10486	2913	16572	8670	16566
	21	8951	1505	45948	23490	5777	24041	16008	28771
	22	16435	1681	46883	45524	10081	31028	21946	47904
	23	10576	1018	18411	34677	5749	6977	10970	28088
	24	3226	636	4060	9265	1781	1210	3236	11356
	25	1087	228	570	2521	437	328	1095	3471
	26	455	137	274	709	230	292	641	2633
	27	392	82	189	822	105	143	227	596
