# Supplementary material for: Pair-barcode high-throughput sequencing for large-scale multiplexed sample analysis
Source: BMC Genomics. 2012 Jan 25;13:43. doi: 10.1186/1471-2164-13-43 (PMC3284879; doi:10.1186/1471-2164-13-43)
Supplement: Additional file 3 — MiRNA read counts of two independent runs. Scatter plots of miRNA expressions of all 32 barcode pairs of two independent runs. [file 1471-2164-13-43-S3.PDF]

Additional file 3, MiRNA read counts of two independent runs.

Scatter plots of miRNA expressions of all 32 barcode pairs of two independent runs. The raw expression value was used and the expression value zero was set as one to draw the scatter plots on logarithmic coordinates.

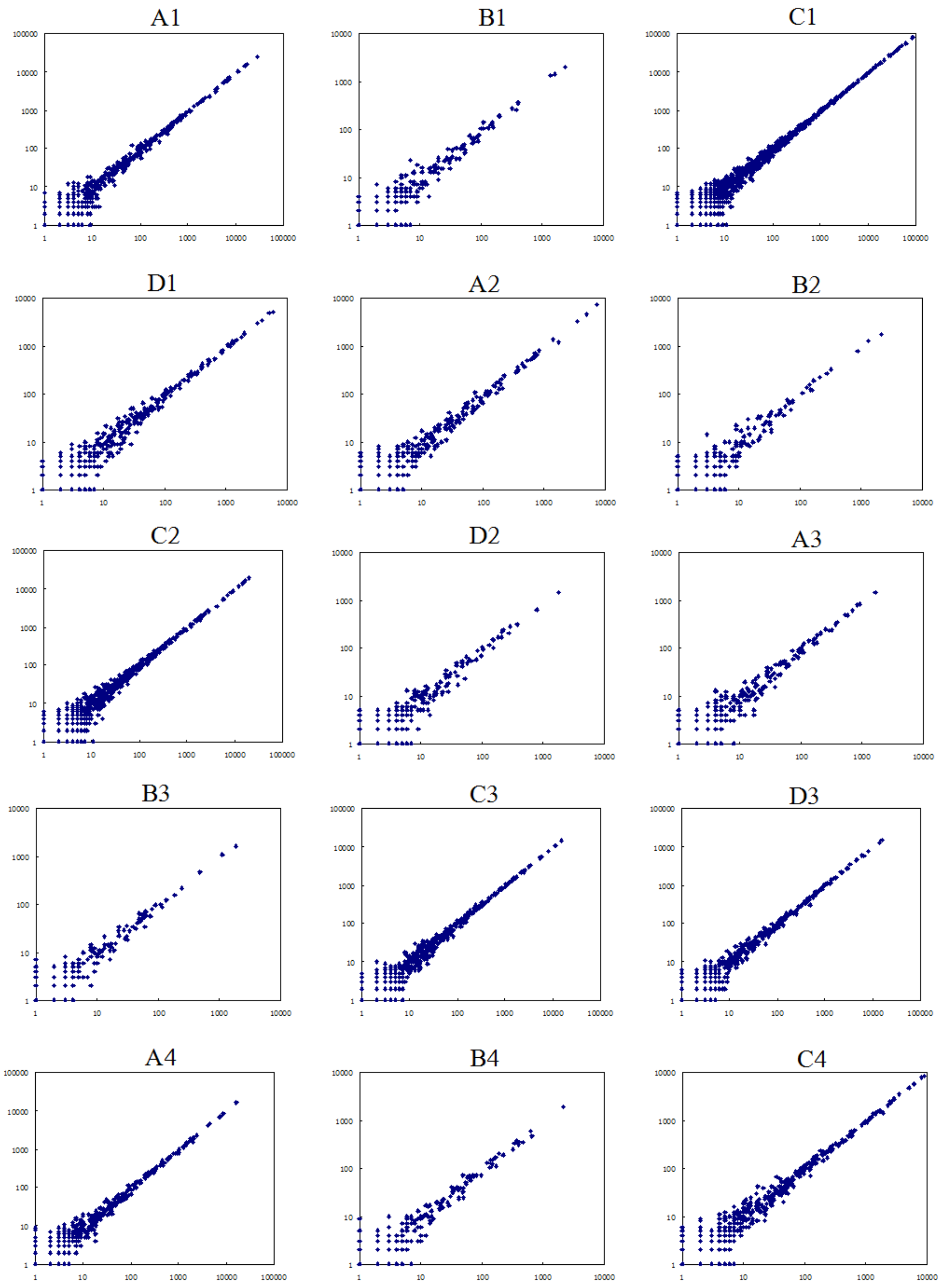

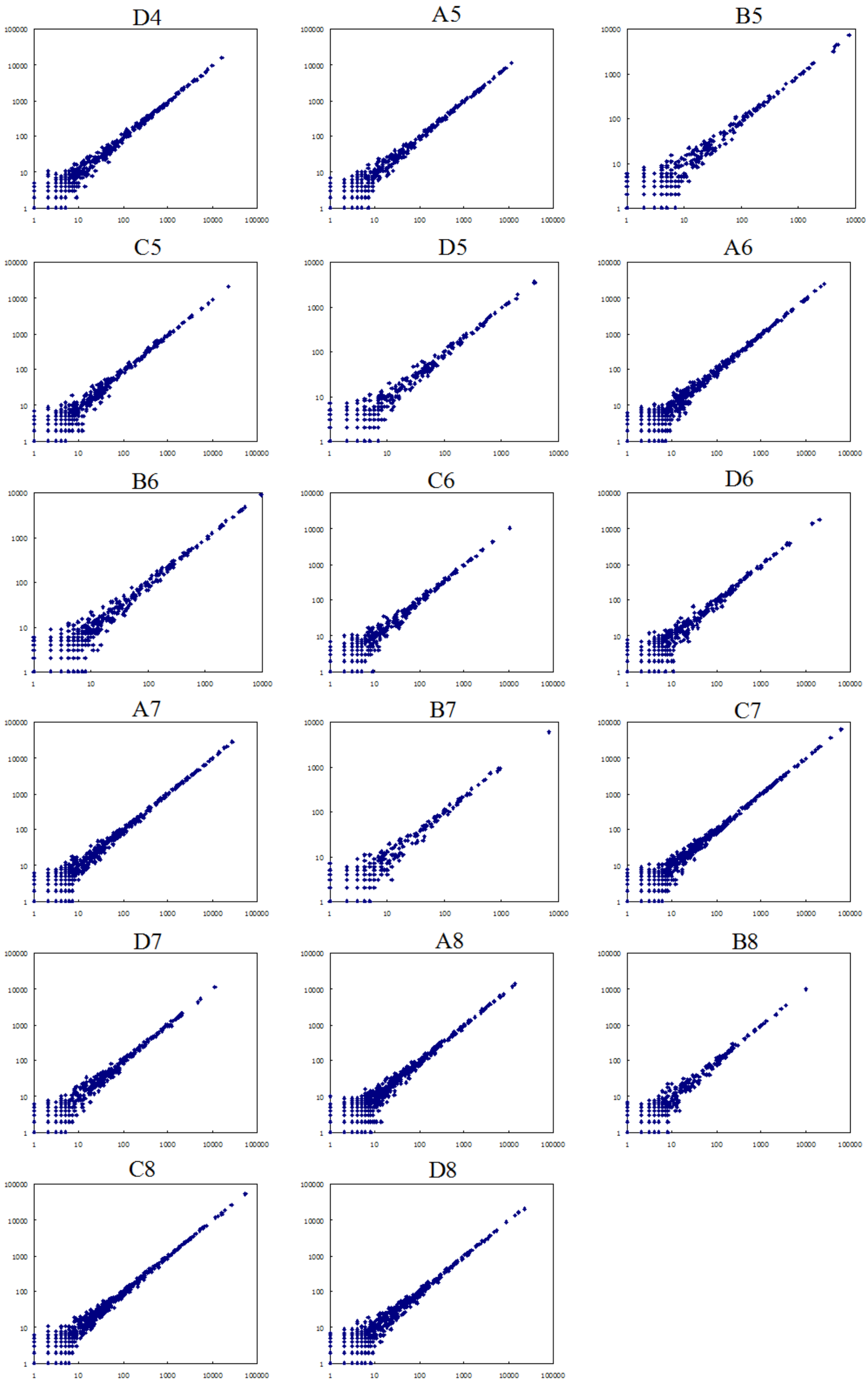
D4
A5
B5
C5
D5
A6
B6
C6
D6
A7
B7
C7
D7
A8
B8
C8
D8
