# Supplementary material for: Pair-barcode high-throughput sequencing for large-scale multiplexed sample analysis
Source: BMC Genomics. 2012 Jan 25;13:43. doi: 10.1186/1471-2164-13-43 (PMC3284879; doi:10.1186/1471-2164-13-43)
Supplement: Additional file 4 — MiRNA read counts of different barcode pairs. Scatter plots of miRNA read counts of the same sample without barcode to with different barcode pairs. [file 1471-2164-13-43-S4.PDF]

Additional file 4, MiRNA read counts of different barcode pairs.

Scatter plots of miRNA read counts of the same sample without barcode to with different barcode pairs. The sample M-14 was selected to evaluate the correlation between the miRNA expression value without barcode and with different barcode pairs. Each figure shows a scatter plot of (logarithmized) raw miRNA expression values determined without barcode (x-axis) vs. (logarithmized) raw miRNA expression values determined with a kind of barcode pair (y-axis). The miRNAs whose raw read counts were >5 in both datasets for comparison were selected to calculate the Pearson’s correlation coefficient.

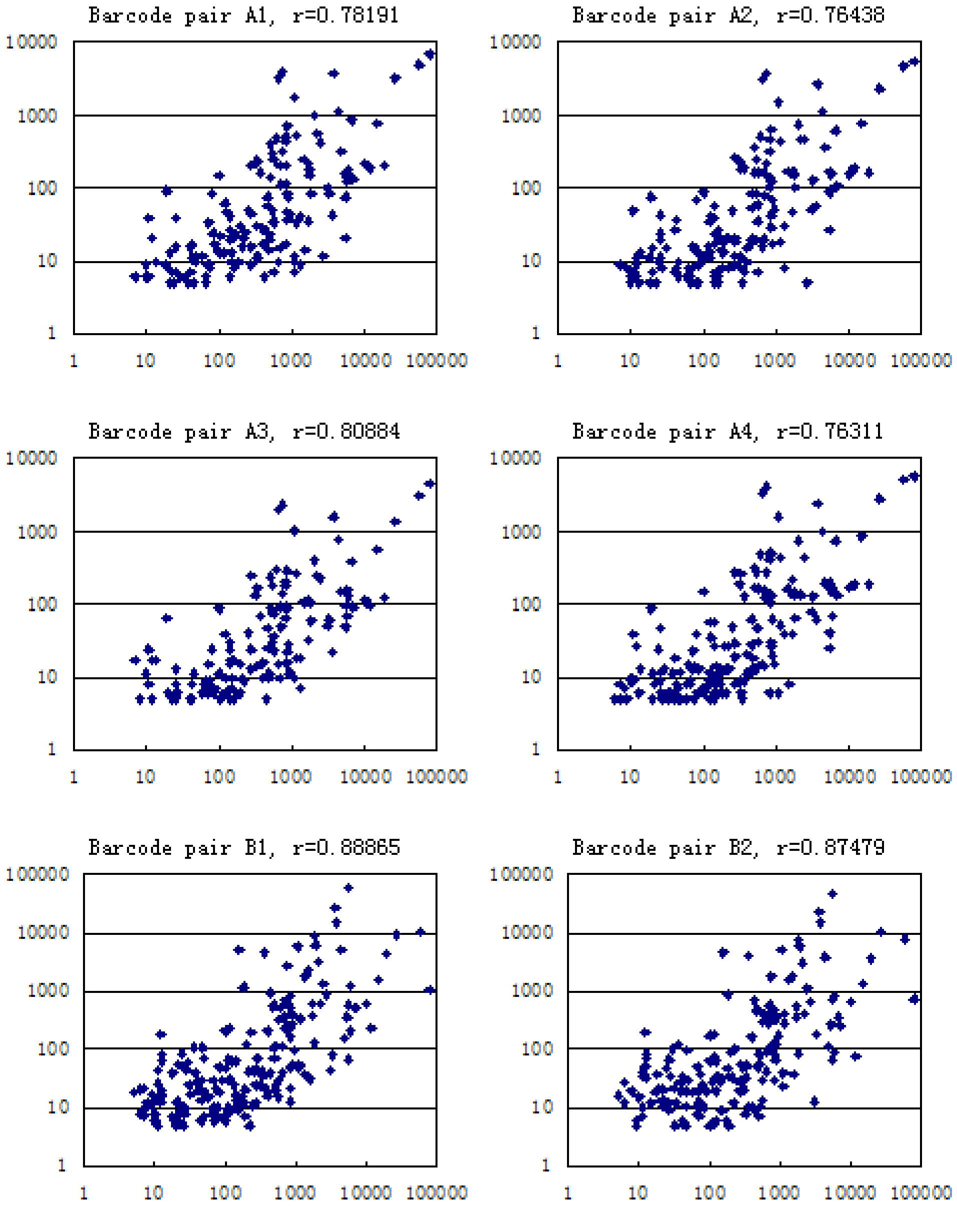

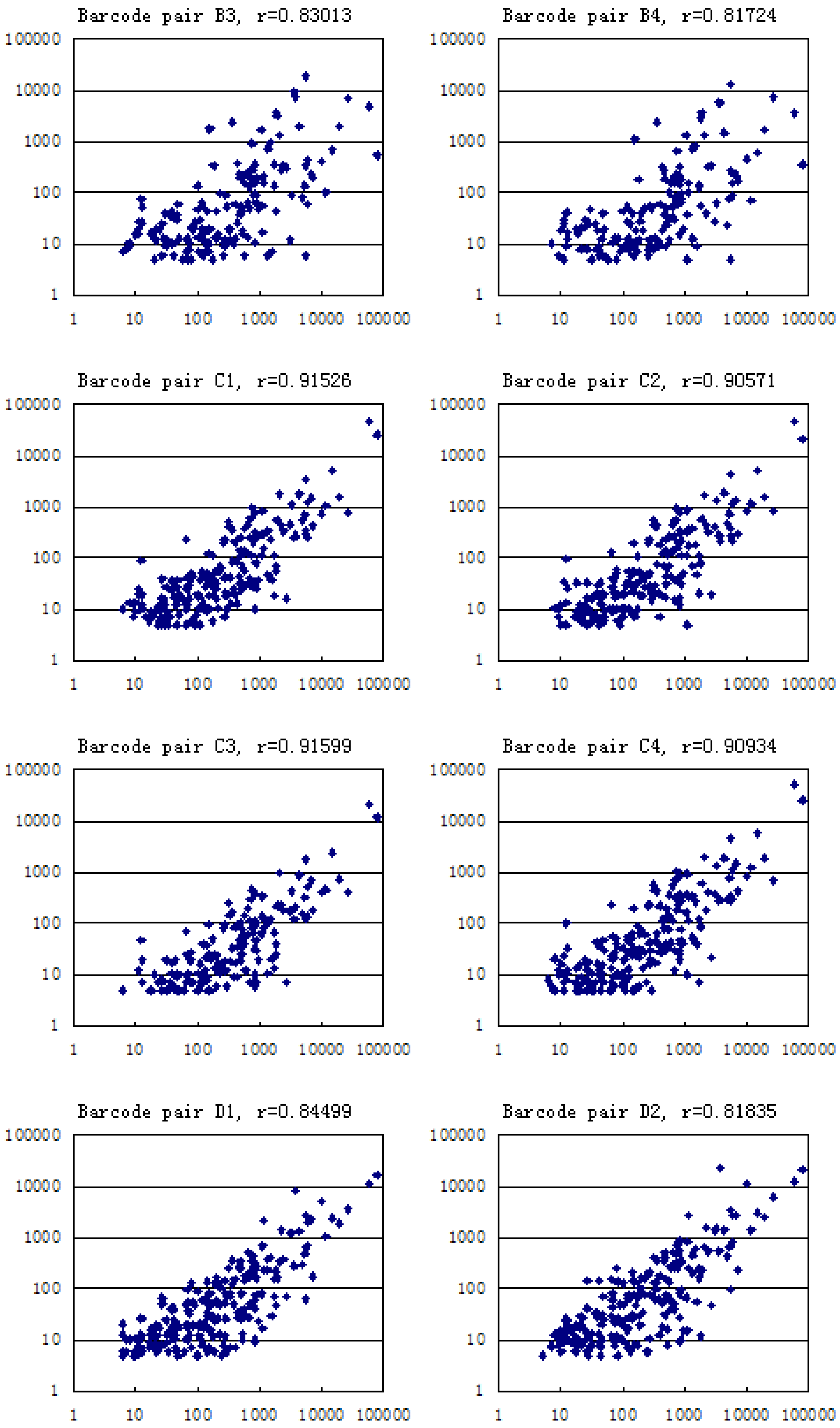

Barcode pair B3, r=0.83013
Barcode pair B4, r=0.81724
Barcode pair C1, r=0.91526
Barcode pair C2, r=0.90571
Barcode pair C3, r=0.91599
Barcode pair C4, r=0.90934
Barcode pair D1, r=0.84499
Barcode pair D2, r=0.81835
100000
10000
1000
100
10
1
1
10
100
1000
10000
100000

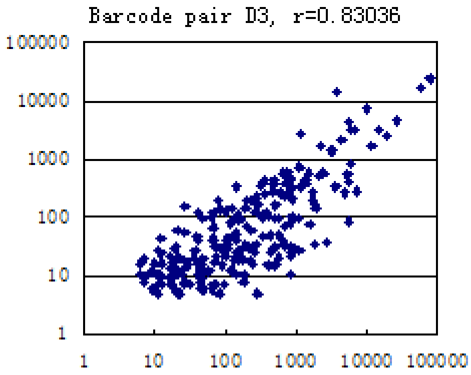
Barcode pair D3, r=0.83036
100000
10000
1000
100
10
1
1
10
100
1000
10000
100000

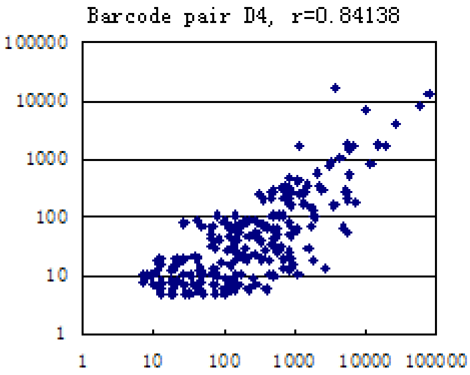
Barcode pair D4, r=0.84138
100000
10000
1000
100
10
1
1
10
100
1000
10000
100000
