# Supplementary material for: Pair-barcode high-throughput sequencing for large-scale multiplexed sample analysis
Source: BMC Genomics. 2012 Jan 25;13:43. doi: 10.1186/1471-2164-13-43 (PMC3284879; doi:10.1186/1471-2164-13-43)
Supplement: Additional file 5 — Highly expressed miRNAs. Highly expressed miRNAs in all 25 datasets. [file 1471-2164-13-43-S5.PDF]

Additional file 5, Highly expressed miRNAs

Table S5 exhibits the highly expressed miRNAs in 25 datasets (19 BC datasets and 6 BO datasets). All miRNAs whose expression value over 100 (after quantile-quantile normalization) in at least 20 datasets are shown in Table S5 (81 miRNAs). The average miRNA expression values of all 25 datasets (row All), 19 breast cancer datasets (row M) and 6 breast carcinoid datasets (row C) are shown in Table S5-3. The miRNAs are ordered by the average expression value in 25 datasets.

Table S5-1

	breast cancer								
	M-1	M-2	M-4	M-5	M-6	M-7	M-8	M-13	M-14
miR-23a	32,994	53,711	15,141	27,246	43,469	22,749	19,619	38,025	26,050
miR-21	21,545	22,714	3,437	8,802	37,415	395	1,293	7,767	10,358
miR-19b	29,943	114,293	25,706	40,252	18,251	29,867	7,802	1,678	1,448
let-7b	4,157	8,705	1,014	8,608	6,048	9,910	1,563	33,655	22,220
miR-27a	1,180	2,317	5,743	5,444	15,829	5,449	10,202	13,093	15,980
miR-1308	9,745	62,447	4,031	3,849	14,131	3,115	4,247	7,817	39,877
miR-181a	13,687	10,241	6,883	4,841	27,870	5,570	12,305	10,629	9,063
miR-29a	1,053	694	6,525	6,729	1,117	5,755	4,103	1,751	1,292
miR-205	5,142	9,875	32,021	16,213	14,146	32,946	10,717	5,403	2,813
miR-1975	3,700	11,115	6,613	6,823	1,546	7,500	794	2,368	5,502
let-7a	805	913	426	3,282	6,551	2,221	986	69,234	20,710
miR-25	12,611	9,243	5,111	7,588	3,302	6,250	2,181	2,853	2,210
miR-26a	2,984	2,106	3,759	12,553	18,106	6,503	5,499	3,749	1,122
miR-99a	351	928	6,823	2,796	141	1,116	351	4,526	781
miR-143	1,700	1,076	2,895	3,285	3,913	1,475	7,629	41,746	23,086
miR-30b	6,911	6,575	8,799	23,303	15,119	44,351	6,234	1,125	1,462
miR-200c	3,484	296	206	220	3,991	569	1,240	448	3,537
miR-451	2,081	2,418	61,913	5,947	592	4,411	5,119	3,730	5,776
miR-23b	4,401	2,738	1,813	3,509	7,866	2,851	4,325	9,472	6,756
miR-375	53,704	4,859	152	7,236	976	10,648	864	571	907
miR-342-3p	7,351	944	5,486	21,588	3,958	3,126	2,211	3,072	2,622
miR-191	12,174	12,745	3,002	9,730	5,502	5,420	10,280	1,467	1,190
miR-10b	276	406	5,571	2,037	317	1,149	455	901	614
miR-199a-3p	1,795	1,716	5,569	5,740	15,322	4,508	980	16,288	10,403
let-7g	437	351	178	549	1,533	392	341	42,747	12,479
miR-125a-5p	7,953	5,351	17,158	23,540	3,092	15,025	1,330	1,239	493
miR-30a	14,277	1,053	2,363	6,126	618	851	989	1,847	1,960
miR-125b	3,700	8,042	27,489	15,397	3,256	6,890	1,674	1,083	283
miR-27b	431	881	1,487	818	2,193	1,379	1,463	992	2,350
miR-103	1,614	2,636	744	1,607	1,532	3,908	669	2,203	1,906
miR-30c	7,304	2,761	7,665	15,360	8,703	7,088	4,889	1,051	1,034
miR-193a-3p	609	1,256	7,523	29,824	503	15,735	4,575	1,989	1,887
miR-1979	482	1,622	1,646	910	1,210	1,096	5,657	6,386	2,557
miR-30e	2,039	819	2,261	2,082	1,984	2,047	2,021	1,006	681
miR-101	307	70	377	437	350	333	315	361	143

miR-181b	1,616	827	2,228	1,125	8,029	1,590	2,213	453	2,449
let-7i	307	343	188	85	897	201	407	12,951	21,792
let-7c	344	406	174	478	1,027	242	337	13,079	2,778
miR-200a	3,130	491	763	3,314	4,495	1,271	2,842	1,573	1,419
miR-320a	2,602	5,593	1,133	932	173	1,272	230	1,472	1,314
miR-146b-5p	1,073	11,053	3,201	2,620	1,398	2,709	4,332	1,746	1,569
miR-185	1,388	835	255	924	212	886	210	15,415	9,811
miR-199a-5p	281	671	866	724	1,655	378	536	1,243	1,273
miR-92a	525	398	2,117	1,918	300	2,554	411	1,248	1,034
miR-10a	96	86	1,643	988	70	382	125	206	178
miR-15b	984	265	2,338	4,178	2,264	3,328	1,501	933	2,323
miR-29c	2,608	1,591	551	3,541	495	3,088	591	242	299
miR-214	435	1,334	894	1,582	1,439	397	364	2,821	977
miR-210	775	21,528	137	203	393	822	175	229	159
miR-200b	643	78	98	183	1,159	146	1,429	626	1,203
miR-151-5p	1,166	577	1,774	2,640	2,691	3,400	1,020	1,760	3,103
miR-145	1,350	1,256	1,305	1,915	2,288	1,032	1,626	2,002	471
miR-141	856	164	748	2,391	2,123	1,670	1,430	201	756
miR-100	58	226	1,084	510	26	187	60	800	151
miR-24	368	476	433	567	933	590	303	782	509
let-7e	303	195	26	197	1,112	212	161	9,723	4,395
miR-148b	763	515	870	2,441	644	1,346	190	846	1,252
miR-222	401	889	1,583	1,596	461	12,807	307	288	73
miR-30d	1,145	351	294	1,125	180	2,959	326	1,577	2,412
let-7f	123	148	161	476	2,291	253	344	10,775	2,374
miR-148a	283	554	3,388	348	1,825	259	888	1,266	490
miR-146a	747	6,770	2,483	1,010	744	2,428	1,314	590	218
miR-29b	907	811	900	677	1,147	844	764	192	162
miR-429	380	78	49	194	899	107	346	133	328
miR-126	101	94	219	192	246	217	719	101	65
miR-34a	1,589	1,802	698	1,225	1,670	1,021	1,501	91	116
miR-423-5p	600	1,396	379	617	347	615	89	741	1,125
miR-223	659	507	4,901	1,655	1,203	2,727	1,957	183	180
miR-150	224	117	1,001	226	198	275	988	773	78
miR-497	257	187	574	325	252	194	299	96	32
miR-106b	447	70	133	66	266	119	165	206	264
miR-151-3p	739	554	819	1,803	928	2,486	1,624	169	420
miR-20a	68	78	422	581	683	837	369	91	848
miR-15a	170	257	1,013	1,323	899	1,378	946	270	436
miR-425	272	265	238	1,001	283	1,265	4,370	443	592
miR-19a	337	1,264	261	610	301	340	119	32	22
miR-423-3p	213	133	122	228	100	393	36	279	240
miR-22	228	445	1166	711	270	467	287	325	363
miR-26b	225	148	114	338	1300	243	336	553	336

miR-424	62	203	195	80	206	151	271	110	113
miR-128	189	55	204	244	244	297	42	192	137

Table S5-2

	breast cancer								
	M-16	M-17	M-18	M-19	M-21	M-22	M-23	M-24	M-25
miR-23a	19,430	16,191	95,730	52,676	36,057	28,420	42,234	72,247	40,998
miR-21	4,519	4,856	32,694	14,846	14,501	9,356	40,985	97,879	3,798
miR-19b	3,139	4,795	5,419	4,017	3,795	17,012	3,946	2,507	2,187
let-7b	10,408	4,313	7,250	6,807	6,556	2,107	5,532	2,723	5,311
miR-27a	23,116	11,756	41,697	25,231	10,431	6,512	7,322	21,394	7,645
miR-1308	16,251	17,050	13,963	12,078	6,600	2,374	1,454	1,165	3,271
miR-181a	8,056	5,604	23,796	15,181	5,055	6,224	4,779	19,772	5,727
miR-29a	17,622	3,315	12,634	11,209	30,806	7,317	22,075	20,260	19,218
miR-205	9,487	5,333	4,564	2,335	13,897	3,579	11,286	9,669	3,376
miR-1975	359	655	1,481	534	2,812	12,090	18,984	18,440	16,282
let-7a	6,422	3,293	6,932	3,421	847	453	3,923	769	2,556
miR-25	2,480	1,884	2,966	2,263	6,729	13,413	14,610	18,594	7,155
miR-26a	10,537	9,853	9,618	5,638	2,893	2,986	4,967	579	3,656
miR-99a	1,460	3,220	1,279	1,353	36,349	3,764	13,036	19,605	7,576
miR-143	613	564	1,239	557	1,220	380	1,026	692	380
miR-30b	1,363	1,203	3,023	876	915	1,052	1,688	563	1,544
miR-200c	13,742	11,889	13,604	18,069	959	1,953	935	1,419	976
miR-451	1,762	1,060	552	570	3,192	4,121	1,112	6,758	5,313
miR-23b	3,243	2,823	8,974	6,176	2,471	3,812	4,146	1,831	4,593
miR-375	640	113	835	398	894	686	696	1,124	940
miR-342-3p	229	710	1,646	568	5,086	8,964	3,233	10,774	6,904
miR-191	1,955	1,940	4,280	1,737	1,038	4,663	4,961	661	1,673
miR-10b	2,296	4,959	1,531	2,354	18,241	6,853	8,474	15,120	4,407
miR-199a-3p	2,480	2,390	2,981	239	3,561	1,545	1,996	4,271	3,993
let-7g	2,678	1,957	2,309	1,708	145	220	844	365	400
miR-125a-5p	225	418	678	205	390	1,612	1,534	561	929
miR-30a	462	1,162	883	745	10,866	777	13,681	4,945	3,667
miR-125b	1,036	1,861	1,172	757	796	332	952	1,016	485
miR-27b	9,650	6,963	3,986	4,861	3,757	3,721	958	2,811	1,760
miR-103	1,993	2,259	2,623	2,934	2,580	11,671	5,982	1,221	6,080
miR-30c	1,370	1,858	1,961	1,044	1,524	519	1,505	532	875
miR-193a-3p	239	214	1,059	241	405	767	456	167	547
miR-1979	529	964	2,690	793	499	622	6,952	1,376	6,851
miR-30e	1,041	2,884	2,109	2,674	5,107	1,337	6,204	6,225	2,695
miR-101	6,596	5,734	4,838	5,434	114	67	103	118	131
miR-181b	1,303	850	1,696	2,871	1,213	4,952	1,163	2,890	1,276
let-7i	755	657	583	804	110	187	297	535	180
let-7c	2,562	1,813	1,002	1,206	683	188	696	229	680

miR-200a	864	1,865	1,587	2,349	861	1,877	4,881	591	1,005
miR-320a	828	798	470	818	1,910	755	508	4,572	1,880
miR-146b-5p	79	84	248	232	2,128	677	2,600	440	1,620
miR-185	504	272	735	442	383	1,080	462	627	324
miR-199a-5p	2,390	2,930	3,703	2,024	1,670	702	1,637	2,677	1,531
miR-92a	355	288	561	1,653	3,729	6,894	2,019	4,132	2,565
miR-10a	178	207	228	210	5,096	5,107	3,239	6,701	1,569
miR-15b	500	1,143	2,201	754	914	1,425	1,910	1,723	1,351
miR-29c	2,554	398	1,657	797	2,174	1,004	2,150	730	2,098
miR-214	933	338	2,364	396	2,217	1,148	2,549	3,093	2,998
miR-210	166	22	89	482	321	1,022	331	805	444
miR-200b	2,588	2,700	1,242	3,000	644	952	1,882	900	1,189
miR-151-5p	354	489	813	899	984	1,536	342	900	985
miR-145	639	535	1,024	407	1,621	652	1,483	980	805
miR-141	439	1,275	1,363	1,006	957	2,982	2,789	458	555
miR-100	196	315	254	209	8,031	879	2,361	4,888	1,107
miR-24	1,767	1,172	2,607	1,121	1,426	1,220	952	1,167	793
let-7e	423	166	1,453	262	159	76	536	352	591
miR-148b	469	649	1,000	550	994	1,848	1,369	2,343	484
miR-222	336	104	409	163	498	51	359	283	458
miR-30d	160	173	357	217	919	965	1,888	1,358	1,687
let-7f	166	202	202	111	193	242	679	247	520
miR-148a	335	386	478	324	1,507	1,038	633	2,114	222
miR-146a	71	100	74	136	109	172	530	1,093	213
miR-29b	1,855	1,055	1,600	985	457	284	844	242	625
miR-429	2,207	2,203	1,527	1,497	173	636	342	62	164
miR-126	2,151	1,104	3,140	776	517	375	536	309	518
miR-34a	309	163	133	171	1,122	348	576	962	362
miR-423-5p	360	422	276	299	442	300	393	219	533
miR-223	194	153	207	132	118	79	114	80	155
miR-150	388	322	618	274	306	448	1,237	684	1,787
miR-497	277	785	387	386	1,924	180	1,226	2,422	1,076
miR-106b	2,004	1,630	909	1,795	58	76	80	113	162
miR-151-3p	30	72	139	112	279	587	570	378	340
miR-20a	606	500	311	1,361	236	350	696	226	756
miR-15a	349	450	378	330	313	119	234	522	155
miR-425	9	19	122	110	148	448	399	352	264
miR-19a	503	280	450	554	254	2,749	262	175	133
miR-423-3p	763	493	576	744	152	400	74	116	220
miR-22	171	91	154	206	467	93	68	879	235
miR-26b	301	253	348	206	130	213	639	33	185
miR-424	285	380	309	246	376	188	399	617	285
miR-128	81	152	172	136	116	284	194	190	396

Table S5-3

	breast cancer	Breast carcinoid						average		
	M-26	C-1	C-2	C-3	C-4	C-5	C-6	All	M	C
miR-23a	33,571	32,108	16,887	23,257	68,681	79,628	19,256	38,255	37,714	39,969
miR-21	42,969	1,553	7,656	8,116	8,558	897	9,768	16,667	20,007	6,091
miR-19b	1,332	2,236	2,969	3,456	6,369	2,938	26,240	14,464	16,705	7,368
let-7b	2,659	113,089	8,236	8,635	17,399	46,604	4,134	13,906	7,871	33,016
miR-27a	7,415	8,064	12,134	17,434	29,109	26,761	5,705	13,479	12,514	16,534
miR-1308	2,079	21,176	17,090	17,166	5,715	4,378	7,023	11,924	11,871	12,091
miR-181a	10,399	3,021	10,422	8,760	9,405	12,511	6,617	10,257	10,825	8,456
miR-29a	27,126	2,620	6,716	9,082	11,887	20,985	3,150	10,202	10,558	9,074
miR-205	3,781	10,142	3,715	3,630	16,491	7,362	5,697	9,745	10,346	7,839
miR-1975	32,044	2,168	348	504	767	3,373	29,103	7,436	7,876	6,044
let-7a	2,576	14,332	4,464	4,821	4,541	17,852	596	7,317	7,175	7,768
miR-25	5,765	3,185	1,627	1,741	1,782	2,333	16,475	6,174	6,695	4,524
miR-26a	4,310	536	7,985	8,735	9,355	10,463	1,368	5,994	5,864	6,407
miR-99a	3,636	12,519	2,113	2,409	3,627	1,463	8,740	5,598	5,742	5,145
miR-143	463	39,449	462	531	2,736	898	382	5,536	4,944	7,410
miR-30b	994	1,039	1,060	934	3,777	2,692	799	5,496	6,689	1,717
miR-200c	809	791	9,077	12,351	8,614	24,749	372	5,372	4,124	9,326
miR-451	827	5,641	859	456	841	1,496	6,351	5,316	6,171	2,607
miR-23b	5,137	2,886	2,602	3,316	8,100	10,411	2,070	4,653	4,576	4,897
miR-375	111	960	660	865	1,138	7,670	9,997	4,306	4,545	3,548
miR-342-3p	6,537	2,434	600	531	1,194	2,558	2,626	4,198	5,000	1,657
miR-191	1,775	1,559	1,678	1,874	2,155	5,236	4,376	4,123	4,536	2,813
miR-10b	2,498	2,603	3,717	3,712	4,050	2,491	7,068	4,084	4,129	3,940
miR-199a-3p	819	1,175	3,032	2,663	4,157	2,727	1,250	4,064	4,558	2,501
let-7g	522	10,475	1,859	1,773	1,822	3,562	366	3,601	3,692	3,310
miR-125a-5p	777	757	284	226	457	258	1,321	3,432	4,343	550
miR-30a	1,402	8,171	864	931	1,795	1,845	1,131	3,336	3,614	2,456
miR-125b	431	1,287	753	1,182	1,441	581	190	3,283	4,034	906
miR-27b	1,017	1,451	5,978	4,017	9,582	4,206	2,386	3,164	2,709	4,603
miR-103	9,385	1,146	1,950	2,296	1,988	1,977	6,180	3,163	3,345	2,590
miR-30c	560	2,643	1,506	1,340	2,929	1,711	575	3,132	3,558	1,784
miR-193a-3p	248	1,135	79	174	330	578	224	2,831	3,592	420
miR-1979	17,493	1,463	552	617	1,071	2,585	2,004	2,745	3,176	1,382
miR-30e	2,269	1,858	2,202	2,235	3,433	2,104	2,064	2,455	2,499	2,316
miR-101	79	339	5,687	5,346	9,178	8,407	100	2,199	1,364	4,843
miR-181b	5,339	1,090	1,849	1,491	1,718	1,430	2,056	2,149	2,320	1,606
let-7i	307	7,595	611	521	777	885	221	2,088	2,189	1,768
let-7c	416	10,836	2,604	1,965	2,616	2,877	279	1,981	1,492	3,530
miR-200a	1,106	813	1,262	1,769	1,685	4,223	464	1,860	1,910	1,703
miR-320a	530	12,468	859	744	1,648	666	567	1,790	1,462	2,825

miR-146b-5p	2,196	983	90	111	350	200	2,726	1,779	2,106	743
miR-185	233	3,258	320	366	584	615	1,294	1,657	1,842	1,073
miR-199a-5p	2,102	768	2,300	2,558	2,636	1,198	2,043	1,620	1,526	1,917
miR-92a	2,381	2,061	266	472	410	549	1,558	1,616	1,846	886
miR-10a	2,650	299	207	196	410	3,383	5,547	1,560	1,524	1,674
miR-15b	1,763	711	1,027	959	874	1,071	641	1,483	1,674	880
miR-29c	1,693	215	969	1,337	2,312	2,523	469	1,443	1,487	1,304
miR-214	2,913	2,078	865	421	641	870	1,632	1,428	1,536	1,084
miR-210	257	113	92	105	264	580	4,179	1,348	1,493	889
miR-200b	1,421	678	2,140	2,421	1,545	3,975	150	1,320	1,162	1,818
miR-151-5p	1,301	774	442	501	671	1,103	1,537	1,270	1,407	838
miR-145	752	2,106	449	424	1,635	697	1,917	1,175	1,165	1,205
miR-141	286	367	821	1,222	1,054	2,061	683	1,146	1,182	1,035
miR-100	834	1,711	202	228	440	199	2,170	1,085	1,167	825
miR-24	818	271	1,221	1,293	2,072	1,641	1,381	1,035	948	1,313
let-7e	538	1,214	381	504	307	1,427	66	991	1,099	650
miR-148b	590	1,801	516	486	637	820	1,358	991	1,008	936
miR-222	241	237	396	184	173	328	319	918	1,121	273
miR-30d	1,233	1,717	126	81	307	304	617	899	1,017	525
let-7f	836	1,107	123	114	317	202	53	890	1,071	319
miR-148a	367	2,360	294	271	257	178	1,076	846	879	739
miR-146a	758	683	68	100	127	41	554	845	1,029	262
miR-29b	836	73	743	987	1,458	2,325	169	838	799	959
miR-429	143	73	1,729	2,167	1,325	2,575	121	778	604	1,332
miR-126	439	68	974	1,251	934	2,302	163	700	622	949
miR-34a	261	282	272	238	477	213	1,432	681	743	486
miR-423-5p	190	4,693	364	388	651	323	709	659	492	1,188
miR-223	169	215	106	122	204	210	50	651	809	151
miR-150	3,956	401	247	213	450	194	525	637	732	338
miR-497	1,023	243	612	711	941	623	361	616	627	582
miR-106b	151	198	1,763	1,639	861	1,429	111	589	459	1,000
miR-151-3p	571	277	25	23	177	95	891	564	664	248
miR-20a	950	226	511	574	477	342	76	487	525	368
miR-15a	190	215	432	397	460	412	79	469	512	333
miR-425	323	243	21	24	70	133	250	467	575	124
miR-19a	133	45	376	433	307	258	804	440	462	370
miR-423-3p	64	152	440	871	520	1,358	95	351	281	573
miR-22	289	209	158	137	187	198	206	309	364	183
miR-26b	403	73	232	301	290	527	55	300	332	246
miR-424	269	56	553	430	367	1304	253	297	250	494
miR-128	304	147	102	109	83	179	274	175	191	149
