# Supplementary material for: Pair-barcode high-throughput sequencing for large-scale multiplexed sample analysis
Source: BMC Genomics. 2012 Jan 25;13:43. doi: 10.1186/1471-2164-13-43 (PMC3284879; doi:10.1186/1471-2164-13-43)
Supplement: Additional file 6 — Mapping and normalization. The detailed progression of mapping and normalization. [file 1471-2164-13-43-S6.PDF]

Additional file 6, Mapping and normalization

1. Mapping

The decoded reads were normalized before mapping. The first 18 colors of the normalized reads were used as seeds in mapping to the other noncoding RNAs, miRBase (Release 14.0) and Human Genome (RefSeq Hg19) consequently, allowing two mismatches. The sequences were extended after the seeds until the adapter sequence was found, allowing 3 mismatches in the remaining 11 colors. All the reads which mapped to the other noncoding RNAs were excluded in mapping to the miRBase (Release 14.0) and Human Genome (RefSeq Hg19). And the reads which mapped to miRBase (Release 14.0) were excluded in mapping to the Human Genome (RefSeq Hg19).

Normalized read:	T01102310230322333112133020103
Seed:	T011023102303223331
Extension:	T01102310230322333112
Adapter sequence:	33020103

After mapping the reads against miRBase(Release 14.0), a read is discarded if it was mapped non-uniquely, or if it does not have a 17nt or longer overlap with a specific mature miRNA. Each remaining mapped read increases the expression count for the miRNA by one. Detail mapping statistics are in supporting material S3.

2. Normalization of the expression count

Quantile normalization is used by Schulte etc. [1] to normalized raw read counts and is shown to be superior to scaling to a given constant. We normalized the raw read counts using quantile normalization. The pure scaling transformations were used to remove a potential bias in miRNA expression across the datasets. By computing the median of differences of corresponding quantile values of a dataset and the reference dataset, the scaling factors were obtained. Dataset A1 was chose as the reference dataset, and miRNAs of absolute count >5 in a dataset and the reference dataset were selected to compute the median of differences of corresponding quantile values.

3. Datasets processing

As the two pilot sequencing runs show high uniform in miRNA expression (Supporting material S5), the corresponding raw read counts obtained in two runs were added together before quantile normalization for further analysis. For the datasets whose read counts of all miRNAs are not large enough, the raw read counts can not response the real expression level of miRNAs. The quantile normalization can not remove the potential bias of the miRNAs across the datasets whose absolute count is zero. For this reason, the datasets of read counts of all miRNAs <50,000 (including dataset M-03, M-09, M-10, M-11, M-12, M-15 and M-20) were discarded before the further analysis. The other 25 datasets were kept for expression analysis.

Reference

1. Schulte, J.H., Marschall, T., Martin, M., Rosenstiel, P., Mestdagh, P., Schlierf, S., Thor, T., Vandesompele, J., Eggert, A., Schreiber, S. *et al.* Deep sequencing reveals differential expression of microRNAs in favorable versus unfavorable neuroblastoma. *Nucleic Acids Res*, **38**, 5919-5928.
