# Supplementary material for: Pair-barcode high-throughput sequencing for large-scale multiplexed sample analysis
Source: BMC Genomics. 2012 Jan 25;13:43. doi: 10.1186/1471-2164-13-43 (PMC3284879; doi:10.1186/1471-2164-13-43)
Supplement: Additional file 7 — Different expressed miRNAs. The 22 miRNAs which were differentially expressed between breast malignant and breast carcinoid based on a t-test. [file 1471-2164-13-43-S7.PDF]

Additional file 7, Different expressed miRNAs.

The 22 miRNAs which were differentially expressed between breast malignant and breast carcinoid based on a *t*-test. This table exhibits the 22 miRNAs which were differentially expressed between breast malignant and breast carcinoid based on a *t*-test (p<0.05). All the miRNAs whose raw expression value over 5 in at least 20 datasets of 25 datasets (19 breast cancer datasets and 6 Breast carcinoid datasets) were evaluated by *t*-test. The miRNAs are ordered by *p*-value.

	BC																			BO						
	M-1	M-2	M-4	M-5	M-6	M-7	M-8	M-13	M-14	M-16	M-17	M-18	M-19	M-21	M-22	M-23	M-24	M-25	M-26	C-1	C-2	C-3	C-4	C-5	C-6	*p*-value
miR-31	19	0	34	11	49	26	7	32	140	362	66	20	186	64	10	0	0	56	51	73	302	281	410	392	21	0.0027
miR-15b	984	265	2338	4178	2264	3328	1501	933	2323	500	1143	2201	754	914	1425	1910	1723	1351	1763	711	1027	959	874	1071	641	0.0118
miR-487b	17	0	28	12	48	6	27	27	22	133	217	91	116	48	54	34	23	25	23	73	222	182	187	50	50	0.0157
miR-101	307	70	377	437	350	333	315	361	143	6596	5734	4838	5434	114	67	103	118	131	79	339	5687	5346	9178	8407	100	0.0172
miR-744	128	0	17	16	6	54	14	23	27	66	36	15	38	7	36	0	0	0	9	79	66	93	33	87	16	0.0186
miR-374a	70	0	56	20	57	4	135	41	0	16	706	826	354	65	217	51	23	16	113	28	1022	680	584	495	92	0.0190
let-7b	4157	8705	1014	8608	6048	9910	1563	33655	22220	10408	4313	7250	6807	6556	2107	5532	2723	5311	2659	113089	8236	8635	17399	46604	4134	0.0202
miR-2110	72	125	30	16	27	49	15	87	102	29	14	39	38	23	77	91	46	51	36	181	25	39	47	116	293	0.0249
miR-21*	62	109	126	217	96	129	85	0	0	23	12	187	82	18	38	108	77	69	155	0	24	31	37	94	42	0.0300
miR-218	26	39	150	133	51	124	79	50	0	139	128	141	75	104	38	34	69	58	60	51	161	122	260	250	61	0.0307
miR-32	132	94	362	165	435	223	223	23	393	19	16	0	16	284	491	97	216	109	129	152	17	20	50	26	153	0.0310
miR-155	83	47	203	44	337	144	457	635	350	130	85	302	119	13	37	165	54	202	512	0	86	112	80	135	92	0.0359
miR-214*	19	39	214	107	66	60	55	23	16	12	11	0	10	181	212	80	62	147	214	45	17	11	0	9	69	0.0360
miR-660	8	0	26	18	29	59	40	46	48	203	147	67	144	158	20	40	314	85	82	147	233	253	167	61	150	0.0360
miR-22	228	445	1166	711	270	467	287	325	363	171	91	154	206	467	93	68	879	235	289	209	158	137	187	198	206	0.0378
miR-342-3p	7351	944	5486	21588	3958	3126	2211	3072	2622	229	710	1646	568	5086	8964	3233	10774	6904	6537	2434	600	531	1194	2558	2626	0.0380
miR-151-5p	1166	577	1774	2640	2691	3400	1020	1760	3103	354	489	813	899	984	1536	342	900	985	1301	774	442	501	671	1103	1537	0.0387

miR-339-3p	139	273	101	80	40	61	31	169	92	8	9	33	12	74	28	114	90	53	72	102	9	10	17	17	34	0.0395
miR-92b	108	39	51	114	58	204	45	233	167	74	58	96	252	126	232	125	252	427	258	79	52	63	153	128	69	0.0431
miR-484	257	125	84	149	136	260	128	101	288	94	69	117	134	146	224	325	211	227	171	51	88	64	143	101	298	0.0467
miR-151-3p	739	554	819	1803	928	2486	1624	169	420	30	72	139	112	279	587	570	378	340	571	277	25	23	177	95	891	0.0482
miR-489	19	0	38	28	27	30	75	64	57	58	7	22	61	29	35	0	49	65	44	113	30	35	294	36	16	0.0496
