# Supplementary material for: Pair-barcode high-throughput sequencing for large-scale multiplexed sample analysis
Source: BMC Genomics. 2012 Jan 25;13:43. doi: 10.1186/1471-2164-13-43 (PMC3284879; doi:10.1186/1471-2164-13-43)
Supplement: Additional file 8 — Clinical data. Clinical data of the patients included in this study. [file 1471-2164-13-43-S8.PDF]

Additional file 8, Clinical data.

Clinical data for the patients included in this study. All the 32 samples were obtained form Wuxi maternal and child care service centre, Jiangsu province, China. The clinical data of these patients are shown in the table below.

No.	Age	Type	Stage	Tumor Size
M1	56	Ductal carcinoma	2	2-5cm
M2	52	Ductal carcinoma	2	>5cm
M3	78	Ductal carcinoma	2	<2cm
M4	44	Ductal carcinoma	3	2-5cm
M5	55	Ductal carcinoma	2	<2cm
M6	46	Ductal carcinoma	2	<2cm
M7	45	Ductal carcinoma	2	<2cm
M8	66	Ductal carcinoma	3	2-5cm
M9	32	Intraductal carcinoma	N/A	>5cm
M10	59	Ductal carcinoma	3	>5cm
M11	59	Ductal carcinoma	3	2-5cm
M12	63	Ductal carcinoma	2	<2cm
M13	44	Ductal carcinoma	2	2-5cm
M14	67	Ductal carcinoma	2	>5cm
M15	52	Ductal carcinoma	2	<2cm
M16	52	Ductal carcinoma	3	2-5cm
M17	44	Intraductal carcinoma	N/A	>5cm
M18	48	Ductal carcinoma	3	2-5cm
M19	54	Ductal carcinoma	3	2-5cm
M20	33	Intraductal carcinoma	N/A	>5cm
M21	70	Ductal carcinoma	3	<2cm
M22	39	Ductal carcinoma	2	<2cm
M23	48	Ductal carcinoma	3	<2cm
M24	44	Ductal carcinoma	3	2-5cm
M25	33	Ductal carcinoma	3	>5cm
M26	46	Ductal carcinoma	3	2-5cm
C1	42	Fibroadenoma	N/A	2-5cm
C2	12	Optimum phyllodes tumor	N/A	>5cm
C3	29	Fibroma	N/A	>5cm
C4	36	Fibroma	N/A	>5cm
C5	44	Adjacent noncancerous	N/A	N/A
C6	44	Atypical hyperplasia	N/A	>5cm
