# Supplementary material for: Pair-barcode high-throughput sequencing for large-scale multiplexed sample analysis
Source: BMC Genomics. 2012 Jan 25;13:43. doi: 10.1186/1471-2164-13-43 (PMC3284879; doi:10.1186/1471-2164-13-43)
Supplement: Additional file 9 — Encoding and decoding. Details of the encoding and decoding progress. [file 1471-2164-13-43-S9.PDF]

Additional file 9, Encoding and Decoding

1. Encoding

We designed a pair of nucleotides barcodes to encode a large scale of samples. The pair of nucleotide barcodes was applied at the two sides of miRNAs. F-barcode was designed in the F-adaptors, and the R-barcode was designed in the R-primers. By generating the sequencing libraries, the pair of barcodes was introduced to the two sides of miRNAs.

In the two pilot sequencing runs, the F-barcode was introduced to the libraries by ligating the F-adaptors to the miRNAs. The 32 miRNA samples were divided into four groups equally and each group was ligated to the one kind of the four F-adaptors. 8 of 32 miRNA samples were ligated to the F-adaptor-A, 8 of 32 miRNA samples were ligated to the F-adaptor-B, 8 of 32 miRNA samples were ligated to the F-adaptor-C, and the left 8 miRNA samples were ligated to the F-adaptor-D. (Table S1.) After the ligation, a size-selection by polyacrylamide gel electrophoresis was operated to purify the library. The R-adaptor was ligated to the all F-adaptor ligated miRNA samples before another size-selection. The R-barcode was introduced to the libraries in PCR reactions after the reverse transcription. For each group of miRNA samples which was divided in the F-adaptor ligation, 8 different R-primers were used to amplify the 8 different libraries. One more size-selection by polyacrylamide gel electrophoresis for each sample was done after the PCR reactions.

Table S1. Oligonucleotide sequences

Oligos	Sequence 5'-3' [a, b]
F-Adaptor-A	p-**AAGCCC**AUCACCGACUGCCCAUAGAGAGG
F-Adaptor-B	p-**GGGCAC**AUCACCGACUGCCCAUAGAGAGG
F-Adaptor-C	p-**CCUGAG**AUCACCGACUGCCCAUAGAGAGG
F-Adaptor-D	p-**AAACGC**AUCACCGACUGCCCAUAGAGAGG
R-Adaptor	CGCCUUGGCCGUACAGCAG
RT-Primer	CCTCTCTATGGGCAGTCGGTGAT
F-Primer	CCACTACGCCTCCGCTTTCCTCTCTATGGGCAGTCGGTGAT
R-Primer-1	CTGCCCCGGGTTCCTCATTCTCT**AAGCCC**CTGCTGTACGGCCAAGGCG
R-Primer-2	CTGCCCCGGGTTCCTCATTCTCT**CACACC**CTGCTGTACGGCCAAGGCG
R-Primer-3	CTGCCCCGGGTTCCTCATTCTCT**CCCCTT**CTGCTGTACGGCCAAGGCG
R-Primer-4	CTGCCCCGGGTTCCTCATTCTCT**CATCGG**CTGCTGTACGGCCAAGGCG
R-Primer-5	CTGCCCCGGGTTCCTCATTCTCT**TCGTTG**CTGCTGTACGGCCAAGGCG
R-Primer-6	CTGCCCCGGGTTCCTCATTCTCT**GGGCAC**CTGCTGTACGGCCAAGGCG
R-Primer-7	CTGCCCCGGGTTCCTCATTCTCT**CCAGAC**CTGCTGTACGGCCAAGGCG
R-Primer-8	CTGCCCCGGGTTCCTCATTCTCT**CTCCGT**CTGCTGTACGGCCAAGGCG

[a] The underline sequences of adaptors are the sequence of F-barcodes, and the underline sequences of primers are the sequence of R-barcodes. [b] The adaptors are RNA oligonucleotides.

The 32 samples were then mixed in a pool at the same concentration to operate emulsion PCR (ePCR). The 32 samples were encoded the combination of F-barcode and R-barcode. The corresponding relation between the barcode pairs and miRNA samples are showed in Table S2. The first two rows exhibit the sequence of the 4 F-barcodes and the first two columns show the sequence of the 8 R-barcodes. The sample in column *I*, row *J*, is encoded by the F-barcode-*I* and

R-barcode-*J*.

Table S2. Barcode pairs combination

		F-barcode			
		A: **AAGCCC**	B: **GGGCAC**	C: **CCUGAG**	D: **AAACGC**
R-barcode	1: **AAGCCC**	A1:M-1	B1:M-9	C1:C-2	D1:C-6
	2: **CACACC**	A2:M-2	B2:M-10	C2:M-16	D2:M-20
	3: **CCCCTT**	A3:M-3	B3:M-11	C3:C-3	D3:M-21
	4: **CATCGG**	A4:M-4	B4:M-12	C4:M-17	D4:M-22
	5: **TCGTTG**	A5:M-5	B5:M-13	C5:M-18	D5:M-23
	6: **GGGCAC**	A6:M-6	B6:M-14	C6:C-4	D6:M-24
	7: **CCAGAC**	A7:M-7	B7:M-15	C7:C-5	D7:M-25
	8: **CTCCGT**	A8:M-8	B8:C-1	C8:M-19	D8:M-26

2. Decoding

Each original SOLiD read was combined by a F3 read and a R3 read. The reads were first decoded by mapping the R3 reads to the 8 R-barcodes in color space allowing one mismatch. In the two pilot sequencing runs, only the first 5 bases were sequenced because of the restriction of the SOLiD multiplexing protocol. The reads were then decoded by mapping the first 6 colors of F3 reads to the 4 F-barcodes in color space allowing one mismatch. Only the reads which uniquely mapped to an R-barcode and an F-barcode were assigned to the correspond dataset. The decoded reads were combined by the remaining 29 colors of the F3 sequence and the last base of the correspond F-barcode. We normalized the decoded reads by transforming all guide bases to thymine and adjust the first color of the reads to make sure that the transformation does not change the true sequence of the decoded reads.

	F3	R3
R-barcode		R-barcode-2 (CCACAC)
R-barcode sequence		G301111
Original read:	T20032031102310230322333112133020103	G30111*
F-barcode sequence	T200320	
F-barcode	F-barcode-A (CCCGAA)	
Decoded read:	A31102310230322333112133020103	
Normalized read:	T01102310230322333112133020103	

In the example above, the R3 sequence of this read mapped to the R-barcode-2, and the first 6 colors of F3 sequence mapped to the F-barcode-A. This read was assigned to the dataset A2. The decoded read was combined by the remaining 29 colors and the last base of the F-barcode-A, adenine. We normalized the decoded read by transforming the guide base adenine to thymine, and adjust the first color from 3 to 0.
